# Proteome analysis provides insights into sex differences in *Holothuria Scabra*

**DOI:** 10.1371/journal.pone.0301884

**Published:** 2024-08-29

**Authors:** Chuhang Cheng, FeiFei Wu, Yizhi Xu, Chunhua Ren, Ting Chen, Shella Li, Peihong Shen, Fajun Jiang

**Affiliations:** 1 Guangxi Key Laboratory of Marine Environmental Science, Guangxi Academy of Marine Sciences, Guangxi Academy of Sciences, Nanning, China; 2 College of Life Science and Technology of Guangxi University, Nanning, China; 3 School of Marine Sciences, Sun Yat-sen University, Zhuhai, China; 4 School of Biological Sciences, University of Edinburgh, Edinburgh, England; 5 CAS Key Laboratory of Tropical Marine Bio-resources and Ecology (LMB) / Guangdong Provincial Key Laboratory of Applied Marine Biology (LAMB), South China Sea Institute of Oceanology, Chinese Academy of Sciences, Guangzhou, China; 6 BASIS International School, Guangzhou, China; Shanghai Ocean University, CHINA

## Abstract

Sex-determining mechanism is still ambiguous for sea cucumber *Holothuria scabra* which only manifests gonochorism in gonad. In this study, proteomic analysis was employed to delineate sex-related proteins and genes in gonads of *H*. *scabra*, subsequently validated through Quantitative real-time polymerase chain reaction (qRT-PCR). A total of 5,313 proteins were identified via proteome sequencing. Among these, 817 proteins exhibited expression in both the ovary and testis, with 445 proteins displaying up-regulation and 372 proteins showing down-regulation (ovary vs testis). Furthermore, 136 and 69 proteins were identified as ovary-specific and testis-specific Differentially Abundant Proteins (DAPs), respectively. And 9 DAP coding genes which play crucial role in ovary and testis were verified by qRT-PCR. Notably, 24 ovary-bias proteins enriched in ribosome pathway strongly indicated the crucial role of ribosome in ovary. This study serves to furnish novel evidence pertaining to sex differences in *H*. *scabra*.

## Introduction

*Holothuria scabra* is one of the nocturnal benthic species that customarily fed on algae and plankton, and it is widely distributed in the tropical waters [1]. Sea cucumbers play significant roles in maintaining the pH balance and alkalinity of the seawater, contributing to the health of coral reef ecosystem [2]. They accelerate bioturbation by ingesting the organic matter in the sediment and dissolve carbonate during feeding, thereby promoting the periodic cycle of calcium carbonate [3]. *H*. *scabra* plays a critical role in nutrient cycling, participating as sedimentary nutrients in the form of food chain [4, 5].

Sexual dimorphism is the defining characteristic of organisms in which male and female reproductive organs occur in different individuals [6]. The sex dimorphism phenotype is thought to be the result of differential gene expression profiles between genders, most prominently in gonads and germ cells. The mechanisms of gender determination and differentiation vary significantly across different metazoans due to repeated, independent lineage-specific evolution and rapid modification of potential molecular pathways [7]. This variation is related to endocrine, neural, environmental, social, and ecological factors, including temperature, season, nutrition, and metabolic substances [8, 9]. To expound upon the molecular mechanisms regulating sexual dimorphism, it is essential to examine the expression patterns of all sex-specific genes, particularly those involved in sexual-biased tissues [10].

Invertebrates display a great variety of different sex-determining mechanisms which implies variety of gene regulated mechanism [11]. Numerous sex-related genes have been identified from a genome-wide scale for the sex differentiation mechanism in insects. Cyclin-related genes and serine/threonine-protein kinases (TSSKs) were suggested to be involved in spermatogenesis, while sex lethal (sxl) and transformer-2 (tra-2) were proven to be associated with sex determination. The roles of ecdysone biosynthesis- and chorion-related genes in oogenesis have been elucidated [12–15]. For Crustacea, differentially expressed genes, such as vitellin, vasa-like and gonadotropin-releasing hormone-like in *Litopenaeus vannamei* [16–18], cyclin A, cyclin B, tra-2 and cell division cycle 2 (cdc2) in *Penaeus monodon* were [19–21], double-sex and mab-3 related transcription factor (dmrt) in *Eriocheir sinensis* [22], proliferating cell nuclear antigen (PCNA) and heat shock protein 90 (hsp90) in *Marsupenaeus japonicas* [23, 24], activated protein kinase C1 (RACK1) and cell apoptosis susceptibility (FcCAS) in *Fenneropenaeus chinensis* [25, 26] were found to be involved in sex determination and differentiation. In shellfish, sex differentiation is affected by double-sex-, soxE, dmrt, β-catenin, forkhead box L2 (foxl2), and foxl2os [27]. In sea urchin *Mesocentrotus nudus*, mitochondrial (trap1) and protein furry homolog-like (fryl) have been found important for sex differentiation [28].

Sex determination mechanism has been studied in sea cucumbers, especially in *Apostichopus japonicus* which is the most studied *Holothurian*, through genome, transcriptome, and proteome [29–32]. For *H*. *scabra*, our laboratory has previously investigated the gender differences by metabolome and sex markers [33, 34]. However, the genetic resources are still scarce and severely hinders the research of sex determination mechanism in *H*. *scabra*. Proteomics presents a promising alternative for the discovery of candidate proteins that exhibit significant differences between genders. Quantitative proteomics has been applied to aquatic animals to analyze crucial proteins and pathways involved in oogenesis and sex reversal [35, 36]. And in sea cucumber, *A*. *japonicus*, has been found gender differences in protein expression profiles of gonad and tube feet [32]. According to proteomics analysis, male *A*. *japonicus* may have an advantage in growth after spawning comparing with females [31]. Thus, in this study, aiming to explore the genetic information associated with sex differences in *H*. *scabra*, we carried out proteomics sequencing analysis of ovary and testis by label-free quantitative proteomics (Labelfree) technology. The goal is to contribute to a comprehensive understanding of proteins expression differences between two sexes and to acquire essential data on reproductive processes in *H*. *scabra*.

## Materials and methods

### Ethical procedures

All experiments and animal treatments were carried out according to the principles of Animal Care and Use Committee of Guangxi Academy of Sciences.

### Samples collection and histological examination

30 wild *H*. *Scabra* with body weigh range from 80 to 120g were collected from Xuwen, Zhanjiang, Guangdong province, China (N20°42′, E109°94′, 29 ◦C). The local seawater conditions were temperature at 29°C, pH at 8.1, and salinity at 30‰. The gonads of the sea cucumbers were promptly sampled and categorized into two parts. Half of the gonads were fixed Bouin fluid for histological analysis, the remaining were the frozen in liquid nitrogen and stored at -80°C for further proteome sequencing. The gonads were fixed in Bouin’s solution for 24 hours, gradually dehydrated using gradient ethanol, clarified with xylene, and embedded in paraffin, paraffin-embedded tissue was sectioned into approximate 0.5cm^3^ cubes and then cut into 5-μm slices by a LEICARM2235 Slice Machine (Leica, Germany). The samples were stained with haematoxylin/eosin (H/E) and sealed with resin. Microscopic observations were conducted on sliced tissues using a Motic BA410 microscope (Leica, Germany) to identify the ovaries and teste. Subsequently, the gonads of 6 females and 6 males) were sent to proteome sequencing.

### Total protein extraction and digestion, Liquid Chromatography-Mass Spectrometry/ Mass Spectrometry (LC-MS/MS)

The gonad tissues from 6 females and 6 males were used to extract protein for LC-MS/MS -based quantitative proteomics analysis, respectively. The tissue sample was pulverized under low temperature and mixed with protein lysis buffer. The resulting solution underwent a series of processes including ultrasonic lysis, DDT red and IAM reaction, acetone precipitation, resuspension, rinsing, and drying. Following this, protein dissolution buffer was added for dissolution, and protein quality tests were conducted using the Bradford Protein Assay Kit.

A 120 μg portion was taken from each protein sample mixed with Protein dissolution buffer TEAB buffer under 37°C, followed by enzymatic cutting and an overnight incubation. The solution was then treated with methanoic acid, centrifuged, and the supernatant was filtered using a C18 desalination column. Rinsing was performed three times with 0.1% methanoic acid and 4% acetonitrile, followed by elution twice with 0.1% methanoic acid and 4% acetonitrile. The eluates were merged, lyophilized, and subjected to LC-MS/MS analysis using the Q Exactive TM HF-X mass spectrometer. Spectrum was searched using PD2.2, Thermo. Inferential statistical analysis was carried out using Mann-Whitney Test for the results of protein assay, and the protein (|log2(fold change) | >3 and p-value < 0.05) with a significant difference in male and female is defined as differentially expressed protein (DEP). Program Interproscan-5 was used for gene ontology (GO) and InterPro (IPR) analysis of the Non-Redundant Protein Sequence Database (including SMART, ProDOM, ProSiteProfiles, Pfam, Panther and PRINTS). At the same time, the co-ortholog group (COG) and KEGG database were used to analyze the protein family and correspondent pathways. Through STRING-db server (http://string.embl.de/), possible protein-protein interaction is also predicted. Pathway enrichment analysis of GO, KEGG and IPR is then carried out.

### Verification of sex differential genes

Total RNA of the whole transcriptome was extracted following the instructions provided in the TRIzol^™^ Reagent (Invitrogen) manual. The process involved tissue homogenization, chloroform extraction of RNA, isopropyl alcohol precipitation, washing with 75% ethanol twice, RNA dissolution in RNase-free H2O after precipitation, and measuring RNA concentration and purity using an Ultramicro spectrophotometer (Nanodrop 2000). Gel electrophoresis was then carried out for the detection of completeness of RNA samples.

For verification, 25 *H*. *scabra* specimens were collected from Dingda Seedling Farm, Wenchang, Hainan province, China (N19°45′, E110°78′) in July 2020. The gonads of these adult sea cucumbers were dissected, and sex identification was performed using the routine wax section method. The RNA extracted from the gonads underwent reverse transcription after concentration adjustment with the PrimeScript^™^ RT reagent Kit with gDNA Eraser (Perfect Real Time) (Takara, Japan).

From the proteome, 9 sex-specific protein encoding genes were randomly selected (primer is shown in Table 1). Following the instructions from SYBP Premix Ex TaqTM II, a 25 μL Fluorescent-Quantitation PCR reaction system was employed (12.5 μL of SYBP Premix Ex Taq (2×), 1 μL of upstream primer, 1 μL of downstream primer, 2 μL cDNA and 8.5 μL RNase-free H2O). The cDNA templates originated from 4 female and 4 male *H*. *scabra*. Thermal Cycler Dice Real-Time System III was used for RT-qPCR with a two-step process. The reaction program included initial denaturation (95°C, 1 min), 40 cycles of 95°C for 5 seconds, and 60°C for 30 seconds, followed by signal collection under 72°C. There were 4 biological replicates and 3 technical replicates each for the genes and β-actin. The relative gene expression was normalized to β-actin by the comparative CT method. Pearson’s r correlation coefficient was calculated to evaluate the correlation between the qRT-PCR and proteomic analysis data [37].

**Table 1:** qRT-PCR primers for genes with significant difference from proteome.

Gene name	Primer sequence (5′ to 3′)
XP_022091644.1	F:CCACCAGTGGGACATACAGT
	R:ACCTCCTCTGCCTCTTGTTC
PIK57317.1	F:GAGGCACAGATGAGCCAAAG
	R:CCGTAATGCAGAGTGTGGTG
XP_011684159.1	F:GTTTGAAGGGCAGTTGCTGA
	R:TGATGCTTCAGGCAACCCTA
XP_022080800.1	F:GCAAAGAAAGGTGGATGGCA
	R:TATCACATGAGCCACAGCCA
XP_011664118.1	F:TTCCGAAACTCGGTCTTCCT
	R:TTCGGCTGGTCCTACCAAAT
XP_022080772.1	F:TGTCGAGTCAAGAACTGCCT
	R:GATGCAGCTAGGGAAGGGAT
AAT01142.1	F:ATTCCTTGCCAGGATGGTCA
	R:TCGTCTGTTCCATCACCACA
XP_022089419.1	F:GCTTGAGCAGTGAGGTTAGC
	R:CTTGGAGAGTTGCAGATGGC
PIK42641.1	F:AGCCTCTTTCTTGGCATCCT
	R:ATGCAGGTCACAGTTGGAGA
β-actin	F:CCAGCATCGTACATCGCAAA
	R:ACTCCGATGGTGGCATTAAA

## Results

### Histological structure of the mature gonad in *H*. *scabra*

**Fig 1:**
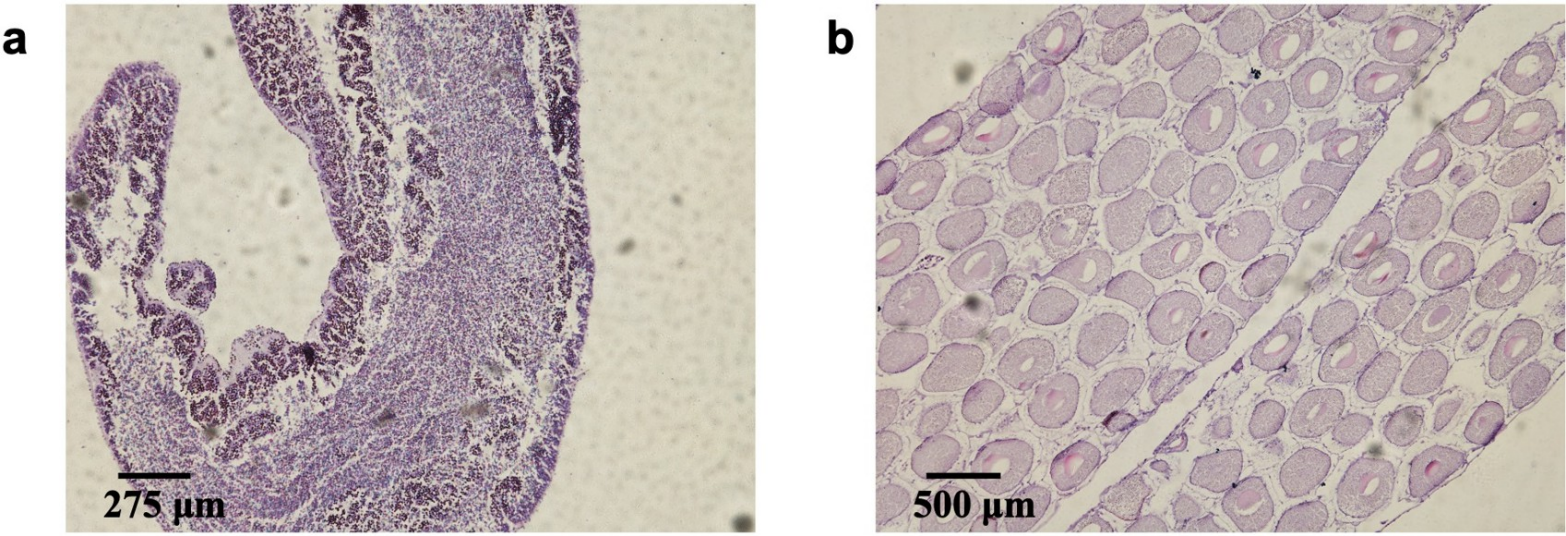
Gonad sections of *H*. *scabra* for sex examination. (a) Testis (b) Ovary.

Total 30 *H*. *scabra* were collected from Xuwen County, Zhanjiang City in September 2019. HE staining was performed to characterize the male and female individuals, respectively. In mature males, the genital atrium was filled with motile sperms which developed from spermatocyte (Fig 1a). Mature ovaries exhibited visible oocytes, forming an irregular polygonal shape due to the cells squeezing each other (Fig 1b). After examined all individuals, 6 mature males and 6 mature females were selected for proteomic analysis.

### Proteomic analysis data

#### Statistics of proteomic analysis data

**Fig 2:**
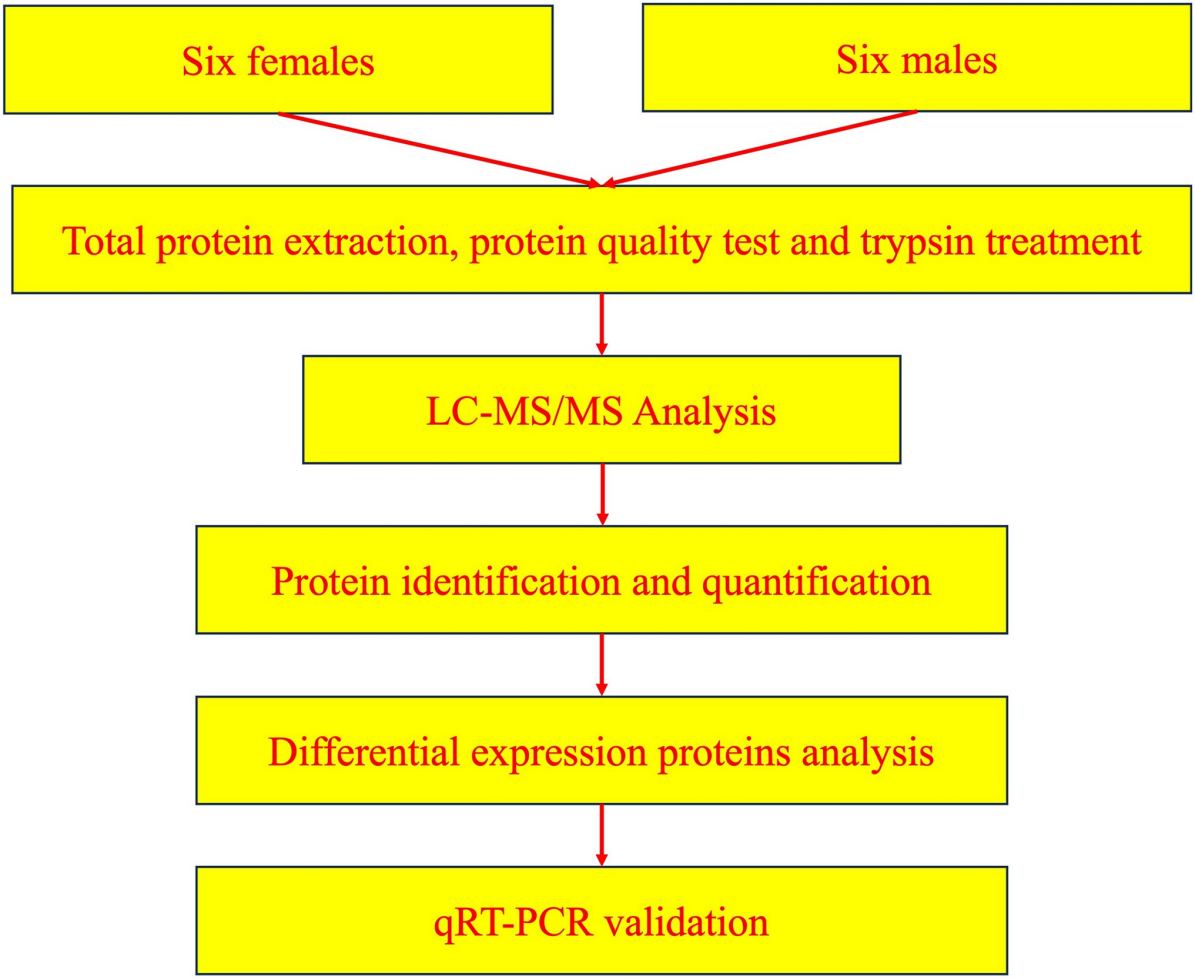
Diagram of workflow for comparative proteomics between two sexes of *H*. *scabra*.

The flowchart of processing steps in our pipeline is shown in Fig 2. Proteome sequencing from selected six male and six female *H*. *scabra* yielded a total of 49,357 unique peptides, resulting in the identification of 5,313 proteins. The distribution of peptide length, protein coverage, and protein mass (S1a, S1b and S1c Fig) demonstrated the accuracy and high reliability of the identification results. The results of Principal coordinates analysis (PCA) showed the significant separation between the proteins of two sexes of *H*. *scabra* (S1d Fig).

#### Functional annotation of all proteins

**Fig 3:**
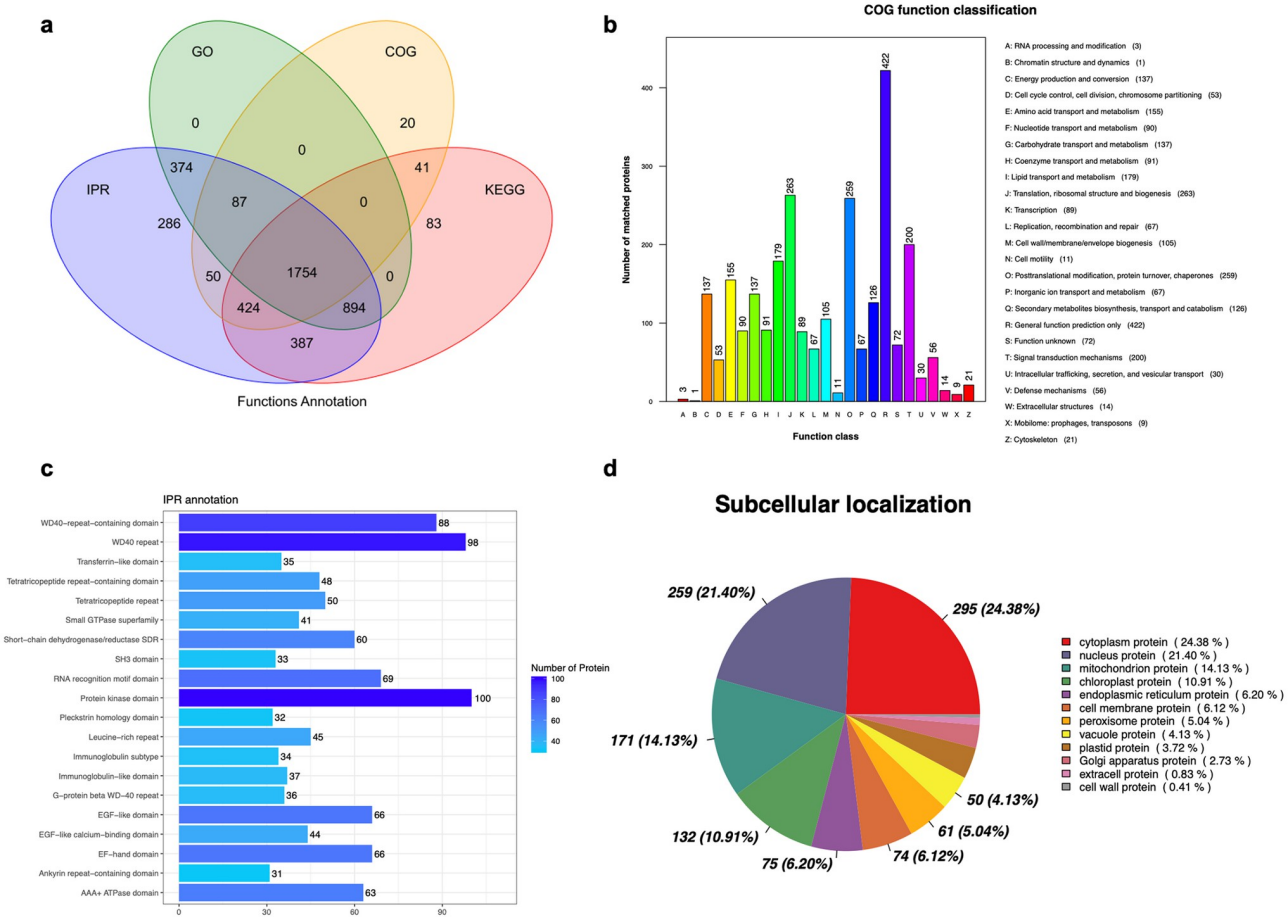
Functional annotation analysis. (**a**) Wayne analysis of annotated proteins using different databases. (**b**) COG functional classification of all matched proteins. (**c**) IPR annotation different analysis of all samples. (**d**) The subcellular localization of all samples. GO gene ontology, COG: Cluster Cluster of Orthologous Groups, IPR: InterPro, KEGG: Kyoto Encyclopedia of Genes and Genomes.

All the quantified proteins were functionally annotated using GO, KEGG, COG, InterPro (IPR), and subcellular localization (Fig 3). The Venn diagram shows a total of 4400 proteins annotated, with proximately 91.1% of them annotated by more than two databases. The GO enrichment analysis demonstrated that most proteins were enriched in molecular function, especially in the terms of protein and ATP bindings (S2 Fig). Furthermore, the KEGG pathway annotation showed that proteins identified in the gonads of *H*. *scabra* were mainly involved in metabolism, including carbohydrate, amino acid, lipid, nucleotide, and energy metabolism as presented in S3 Fig. The COG analysis classified the proteins into 26 functional categories including translation, ribosomal structure, biogenesis, posttranslational modification, protein turnover, chaperones, and signal transduction mechanisms (Fig 3b). IPR annotation analysis mainly identified protein kinase domain, RNA recognition motif domain, and WD40 repeat-containing proteins (Fig 3c). Subcellular location analyses were performed that cytoplasmic proteins (24.38%) and nucleus proteins (21.40%) comprised the largest proportion among the total proteins (Fig 3d).

#### Analysis of the DAPs associated with GO and KEGG pathways

|log2(fold change) | >3 and p-value < 0.05 were set as a threshold to identify Differentially Abundant Proteins (DAPs). A total of 817 DAPs including 136 ovary-specific proteins and 69 testicle-specific proteins were obtained in samples after comparative analyses. Compared to the testis, there were 445 upregulated DAPs and 372 downregulated DAPs in the ovary (Fig 4a). Fig 4b showed significant difference between female and male gonads and the consistency of DAPs among every sample, which demonstrated the reliability of the data. Furthermore, the top 11 up-, and downregulated DAPs coding genes between two sexes were shown in S1 Table (p-value<0.001). And Table 2 listed 25 genes associated with sex and gametogenesis including egg coat matrix protein, egg binding receptor protein 1 precursor, sperm flagellar protein and other sperm-associated proteins which were participated in the structure of gametes.

**Fig 4:**
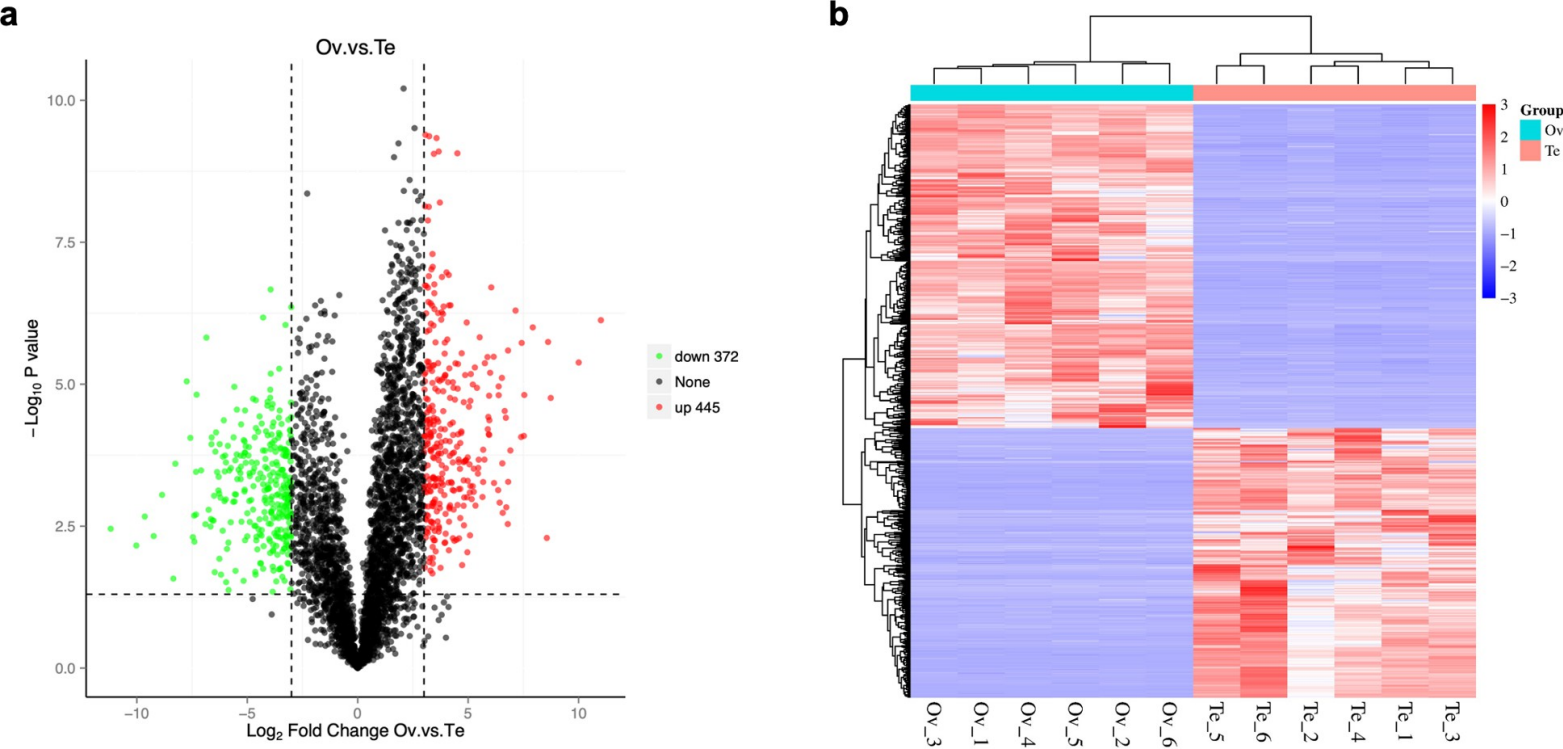
Differential expression analysis of proteins between ovary and testis. (a)Volcano plot (b) Heatmap.

**Table 2:** The list of sex and gametogenesis related DAPs between ovaries and testis.

Gene	Description	log2FC	Trend	Pvalue
**BAJ41225.1**	egg coat matrix protein	8.616619037	up	1.80E-06
**BAJ41227.1**	egg coat matrix protein	7.924463339	up	9.99E-07
**BAJ41226.1**	egg coat matrix protein	7.423074104	up	1.88E-06
**AAP44488.1**	egg binding receptor protein 1 precursor	3.301188988	up	3.76E-07
**PIK35444.1**	sperm flagellar protein 1-like	-3.022187941	down	5.28E-04
**XP_022090776.1**	cilia- and flagella-associated protein 44-like isoform X3	-3.266191646	down	1.44E-04
**XP_022105499.1**	cilia- and flagella-associated protein 57-like	-3.362091033	down	1.72E-03
**PIK57014.1**	primary ciliary dyskinesia protein 1-like	-3.456240939	down	1.35E-03
**PIK42503.1**	cilia- and flagella-associated protein 70	-3.641234272	down	2.65E-04
**XP_020604067.1**	cilia- and flagella-associated protein 20	-3.892563548	down	2.65E-05
**XP_022107058.1**	cilia- and flagella-associated protein 157-like	-3.912204831	down	1.80E-03
**XP_022104631.1**	sperm-associated antigen 16 protein-like	-4.063316144	down	5.93E-04
**PIK44114.1**	testis-expressed sequence 43 protein-like	-4.072539088	down	7.95E-05
**PIK59902.1**	sperm-tail PG-rich repeat-containing protein 2	-4.259111937	down	7.12E-03
**XP_022086482.1**	cilia- and flagella-associated protein 58-like	-4.2825065	down	5.77E-04
**PIK58729.1**	cilia- and flagella-associated protein 53	-4.444913361	down	2.40E-03
**PIK44983.1**	sperm-associated antigen 6	4.739561975	down	3.51E-04
**XP_022102412.1**	dynein intermediate chain 3, ciliary	-4.947083538	down	1.80E-04
**PIK34818.1**	testis-expressed sequence 36 protein-like isoform X1	-5.533452907	down	1.94E-02
**XP_022081058.1**	sperm-tail PG-rich repeat-containing protein 2-like isoform X1	-5.845874193	down	4.22E-02
**XP_022108113.1**	testis, prostate, and placenta-expressed protein-like	-6.612017698	down	8.66E-05
**PIK36385.1**	63 kDa sperm flagellar membrane protein	-8.346589397	down	2.65E-02
**XP_787834.2**	testis-specific serine threonine-protein kinase 1-like	testis-specific	down	0.00E+00
**XP_022091644.1**	cilia- and flagella-associated protein 46-like isoform X7	testis-specific	down	0.00E+00
**PIK57317.1**	sperm-associated antigen 8-like	testis-specific	down	0.00E+00

**Fig 5:**
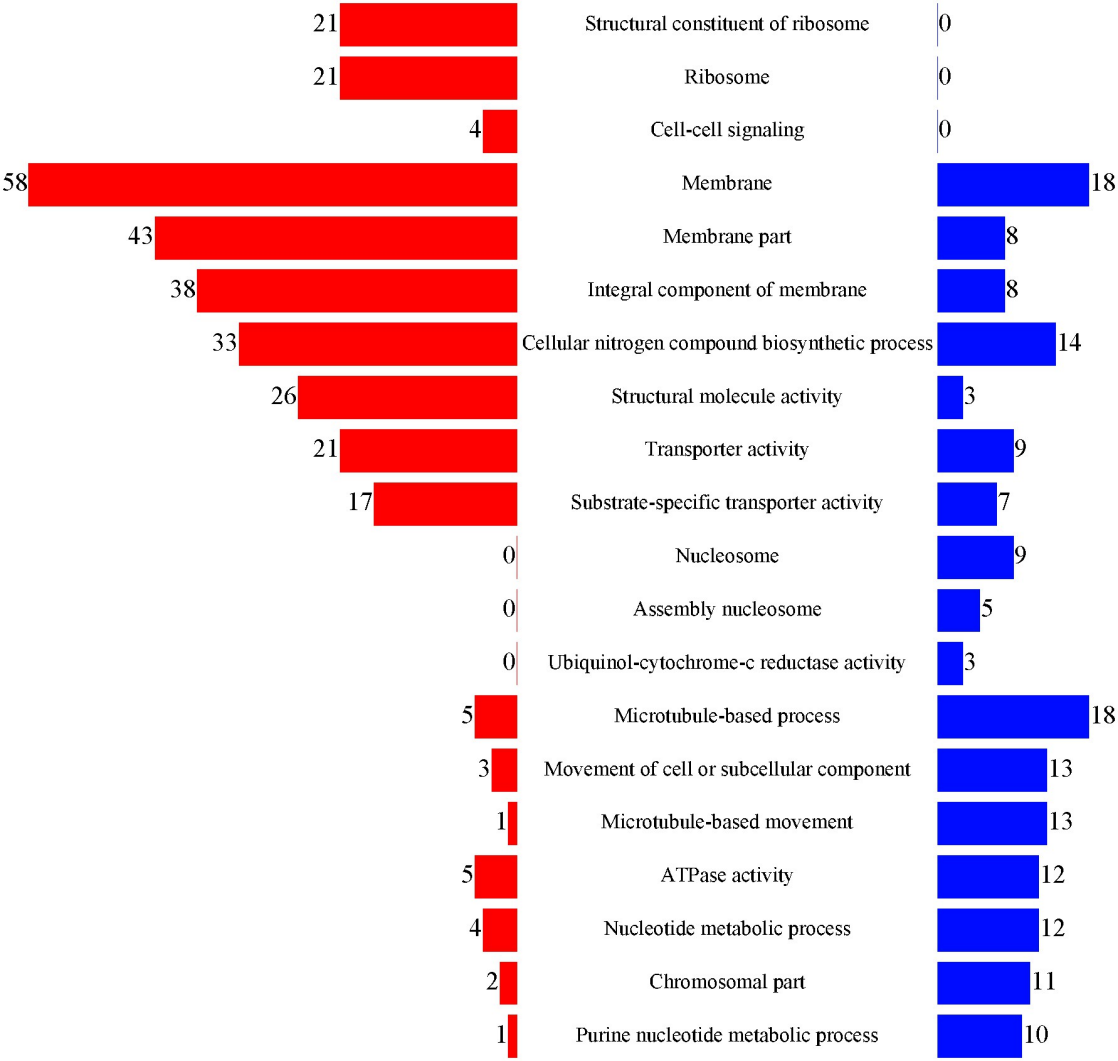
Number of DAPs in the 20 enriched GO terms (P<0.05). Red and blue color represents up-regulated and down-regulated proteins, respectively (Ov vs Te).

Using gene ontology (GO), all DAPs were matched to 403 GO terms. The GO annotation chart showed the 29 enriched GO terms which categorized into three functional groups (S4 Fig). Fig 5 shows the top 10 ovary-bias and the top 10 testis-bias functional terms (P<0.05). Among them, Structural constituent of ribosome and Ribosome enriched 21 and 21 female-specific proteins like 40S ribosomal proteins and 60S ribosomal proteins such as L3-like, L8, S6 and RPL15, which indicated strong ribosome-related activities in ovary. The GO terms related to membrane, membrane parts, and integral components of the membrane were enriched in many proteins upregulated in females. Most of upregulated proteins in testis were enriched under GO terms related to microtubule movement like microtubule-based process and microtubule-based movement. That is because the movement of sperms depends on the activities of flagella and the mature testicles are full of sperms. Proteins associated to nucleosome and assembly nucleosome like histone H1, H3, H4-like and H5 were significantly high expression in testis. Ubiquinol-cytochrome-c reductase activity and ATPase activity, which involves in energy metabolism, were enriched many testis upregulated proteins.

**Fig 6:**
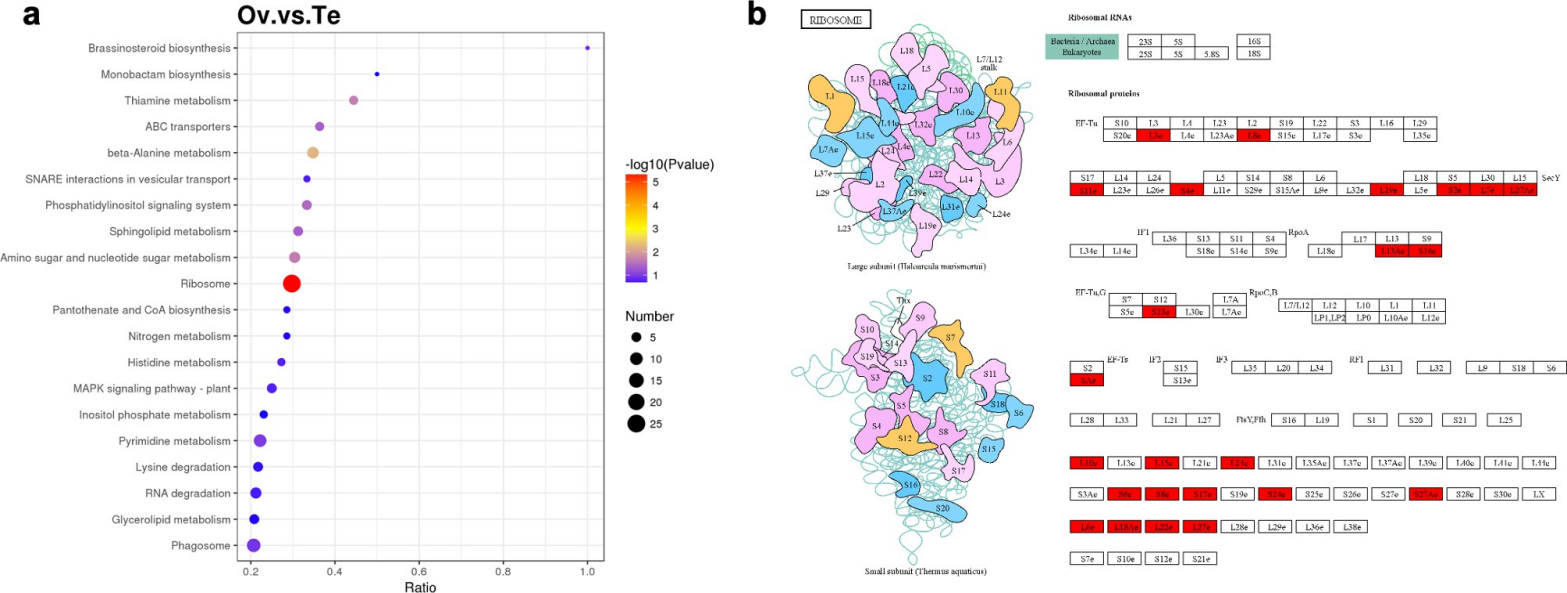
KEGG enrichment analysis. (a) The differentially abundant proteins between ovary and testis. (b) Ribosomal pathway diagram. Red color represents up-regulated proteins.

The KEGG analysis showed that differential proteins enriched the pathways associated with biochemical metabolic and signal transduction including sphingolipid metabolism, ABC transporters, phosphatidylinositol signaling system, amino sugar and nucleotide sugar metabolism, thiamine metabolism, beta-Alaine metabolism, and ribosome (Fig 6a). Notably, the ribosomal pathway enriched 24 up-regulated proteins which have similar results in GO annotations (Fig 6b).

### Validation of gene expression from proteome by qRT-PCR

**Fig 7:**
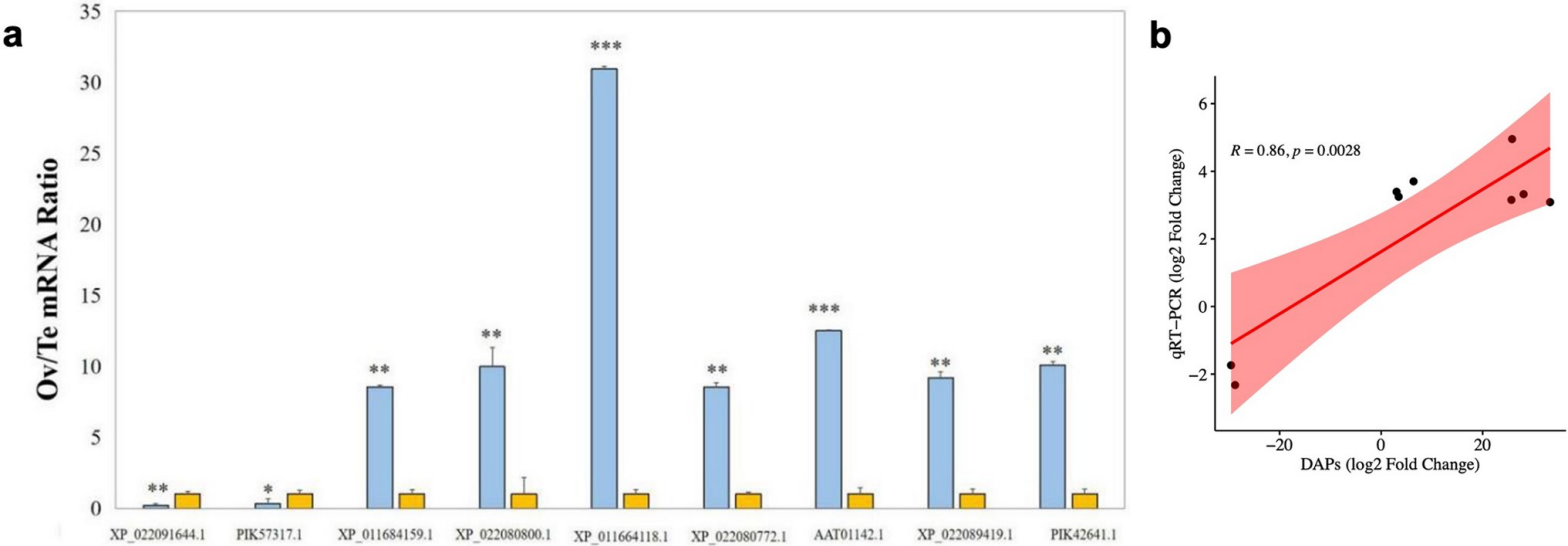
Quantitative real-time PCR (qRT-PCR) results for 9 coding genes. (a) Relative expression levels of genes encoding DAPs. Y-axis denotes the fold change in gene expression of Ov/Te and all expression levels in testis are set to 1. Blue represents Ovary, yellow represents Testis. (b) Pearson’s correlation analysis of qRT-PCR and proteomic data for DAPs.

Totally, 75 sex-related genes were filtered according to the GO, KEGG annotation and Ov/Te expression pattern (shown in S2 Table) and 9 of them were randomly selected analyze by qRT-PCR. As the results shown in Fig 7a and Table 3, seven of the genes in ovaries were verified significantly upregulated, and 2 of the genes were significantly downregulated. A strong correlation of qRT-PCR and proteomic analysis data was shown (R = 0.86, Fig 7b), indicating the reliability of label-free quantitative proteomics analysis to investigate the protein expression profiles of sex difference in *H*. *scabra*.

**Table 3:** List of 9 sex differential genes after qRT-PCR verification.

Gene	Description	Trend
**XP_022091644.1**	cilia- and flagella-associated protein 46-like isoform X7	down
**PIK57317.1**	sperm-associated antigen 8-like	down
**AAT01142.1**	proteoliaisin	up
**XP_022089419.1**	laminin subunit alpha-like isoform X2	up
**XP_011684159.1**	hyalin	up
**XP_022080800.1**	phosphatidylinositol 4-kinase type 2-beta-like	up
**XP_011664118.1**	alpha-N-acetylgalactosaminidase isoform X2	up
**XP_022080772.1**	STE20-like serine threonine-protein kinase isoform X1	up
**PIK42641.1**	double-stranded RNA-binding protein Staufen-like 1 isoform X2	up

## Discussion

The sea cucumber *Holothuria scabra* is an economically important species of echinoderm in Asian market because of its high nutritional, pharmaceutical and economic value [38]. The aquaculture of *H*. *scabra* is also popular for meeting the increased demands of market consumption and natural stock restoration [39]. During sea cucumber culturing, usage of clear sexes parents will be benefit for the process of breeding and reproduction. However, the sexes of *H*. *scabra* cannot distinguish from appearance which may hinder the aquaculture of this species. And the sex determination of holothurians is still ambiguous. Previously, we have investigated the metabolomics profiles and sex makers of two sexes [33, 34]. In this study, to enrich our knowledge of sex difference of *H*. *scabra*, the comparative proteomics between ovary and testis was performed using label-free quantitative method.

Proteoliaisin is a protein that participates the assembly process of fertilization envelope in sea urchin [40]. In echinoderms, proteoliaisin interacts with another protein ovoperoxidase to form a 1:1 complex. This complex inserts into the fertilization envelope to mediates hardening of the assembled envelope [41]. In ovary of *H*. *scabra*, ovoperoxidase and proteoliaisin were significantly upregulated, indicated that the key components of formation of the fertilization envelope mainly exist in ovary and would interact after fertilization. Interestingly, hyalin, a large glycoprotein in the hyaline layer, was also have higher expression level in ovaries. The hyaline layer locates underneath the fertilization envelope in zygote and play a role in blocking against polyspermy [42]. Hyalin is also involved in regulating adhesive relationships as a specific cell adhesion molecule in the developing sea urchin embryo [43]. Laminin α subunit, upregulated in ovary, can assemble into various laminin isoforms and is crucial for protein correct localization in the development of *Caenorhabditis elegans* [44]. Those evidence suggested that the eggs carry many important proteins as preparation for early embryonic development in *H*. *scabra*. In sperm, cilia- and flagella-associated protein (cfap) family and sperm-associated antigen 8 (spag8) are essential component of microtubule doublets (DMTs) which are structural blocks of tail or flagella [45, 46]. Significantly upregulated in male’s gonad, cfap and spag8, played roles in spermatogenesis including sperm motility and microtubule formation to ensure the viability of sperm.

Eukaryotes 80S ribosomes consist of a small (40S, including an 18S RNA and 33 proteins) and large (60S, including 25/28S, 5.8S, 5S rRNA and 49 proteins) subunit [47]. Interestingly, many rRNA and ribosomal proteins have been linked with the ovarian development of aquatic animals. Previous studies in fish and reptiles have found the overwhelming accumulation of 5S rRNA in ovaries which indicated that its crucial role in oocytes [48–50]. And 5S/18S rRNA ratio can serve as markers to distinguish sexes in fish [51]. Ribosome protein S24 has been demonstrated as a potential stimulator in promoting the development of ovaries in east Asian river prawn *Macrobrachium nipponense* [52]. Moreover, during oogenesis in the sea urchin *Paracentrotus lividus*, the expression of ribosomal protein S24 (RPS24) is increased [53]. Ribosome proteins including L3, L8, L10, L15, S11, S4, S23, S24, S16 only upregulated in females which indicated that the ribosome plays a crucial role in ovary. The oocytes accumulate reserve substances for proper development of the embryo and ribosome contribute considerably to the synthesis of proteins in this process.

In mammals, successful production of mature sperm involves the process of chromatin organization which make itself become highly compacted in the sperm head [54]. Chromatin remodeling of the male genome during spermiogenesis relies on nucleosome. A nucleosome consists of a section of DNA that is wrapped around a core of histone proteins which is the basic repeating subunit of chromatin. During spermiogenesis, nucleosome transfer from a histone-based structure to a mostly protamine-based configuration which lead the chromosomes to become compact and condensed [55, 56]. Functional proteins of nucleosome and assembly nucleosome including histone H1, H3, H4-like and H5 were significantly high expression in testis. That result demonstrated those proteins is crucial to generating a viable male gamete in *H*. *scabra*. Motility and morphology are also thought to be indispensable for the fertilizing ability of sperm. Microtubule-based processes in spermatogenesis involve in sperm head shaping and sperm flagella development [57]. Thus, it can be understood that sex differential proteins related to microtubules are enriched in testis.

The utilization of label-free quantitative proteomics allowed us to conduct a comparative proteomics analysis between two sexes of *H*. *Scabra* and to investigated protein candidates that might be involved in sex differences. In present study, we identified and verified 2 downregulated and 7 upregulated genes which involve in sperm motility and assembly process of fertilization envelope respectively. According to functional analysis, ribosomal proteins, membrane proteins, membrane part proteins and integral component of membrane were upregulated in ovary proteome while nucleosome, assembly nucleosome, microtubule movement, ubiquinol-cytochrome-c reductase activity and ATPase activity related proteins were high expression in testis. Notably, ribosome pathway only enriched 24 ovary-bias proteins which strongly indicated the crucial role of ribosome in ovary. Overall, our proteome results provide a novel insight for the study of sex mechanism in *H*. *Scabra*.

## Supporting information

S1 Fig Statistics of the quantitative proteomics data of different samples. (a) The distribution of peptide lengths in all samples. (b) The distribution of peptide coverage in all samples. (c) Numbers of proteins with different masses in all samples. (d) Principal coordinates analysis of four types of individuals. PC: principal coordinate.
(PNG)

S2 Fig GO enrichment analysis of labeled proteins. (PNG)

S3 Fig KEGG enrichment analysis of labeled proteins. (PNG)

S4 Fig GO enrichment analysis of abundant proteins in two comparison groups. (PNG)

S1 Table 22 of the corresponding genes for significantly difference proteins between ovaries and testis. (XLSX)

S2 Table Selected proteins list for qRT-PCR validation. (XLSX)

S3 Table Pearson correlation coefficient analysis between qRT-PCR and proteomic analysis of 9 verified genes. (XLSX)
