# Supplementary figures and images for: Proteome analysis provides insights into sex differences in Holothuria Scabra
Source: PLoS One. 2024 Aug 29;19(8):e0301884. doi: 10.1371/journal.pone.0301884 (PMC11361572; doi:10.1371/journal.pone.0301884)

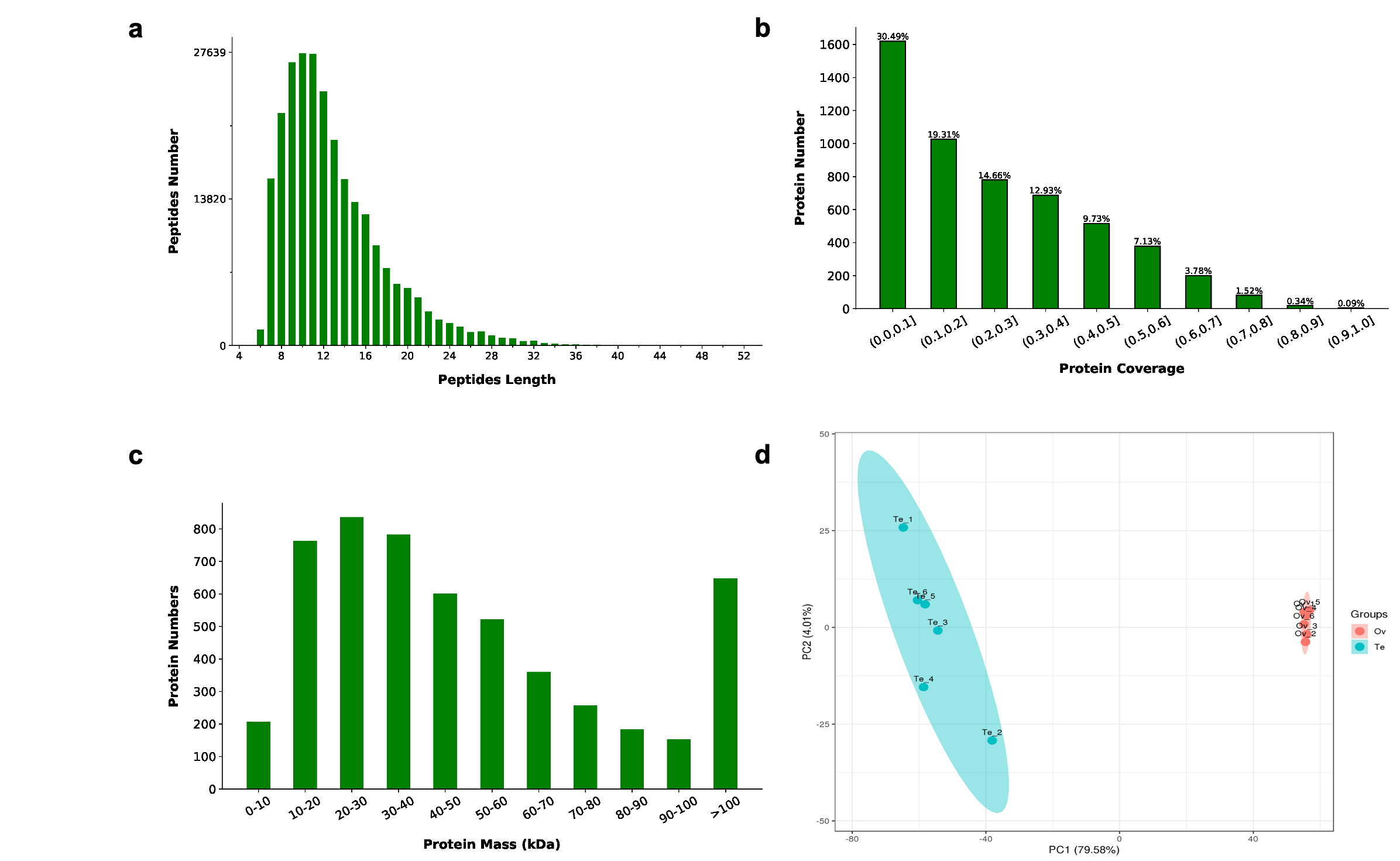

Supplement: S1 Fig — (a) The distribution of peptide lengths in all samples. (b) The distribution of peptide coverage in all samples. (c) Numbers of proteins with different masses in all samples. (d) Principal coordinates analysis of four types of individuals. PC: principal coordinate. (PNG) [file pone.0301884.s001.png]

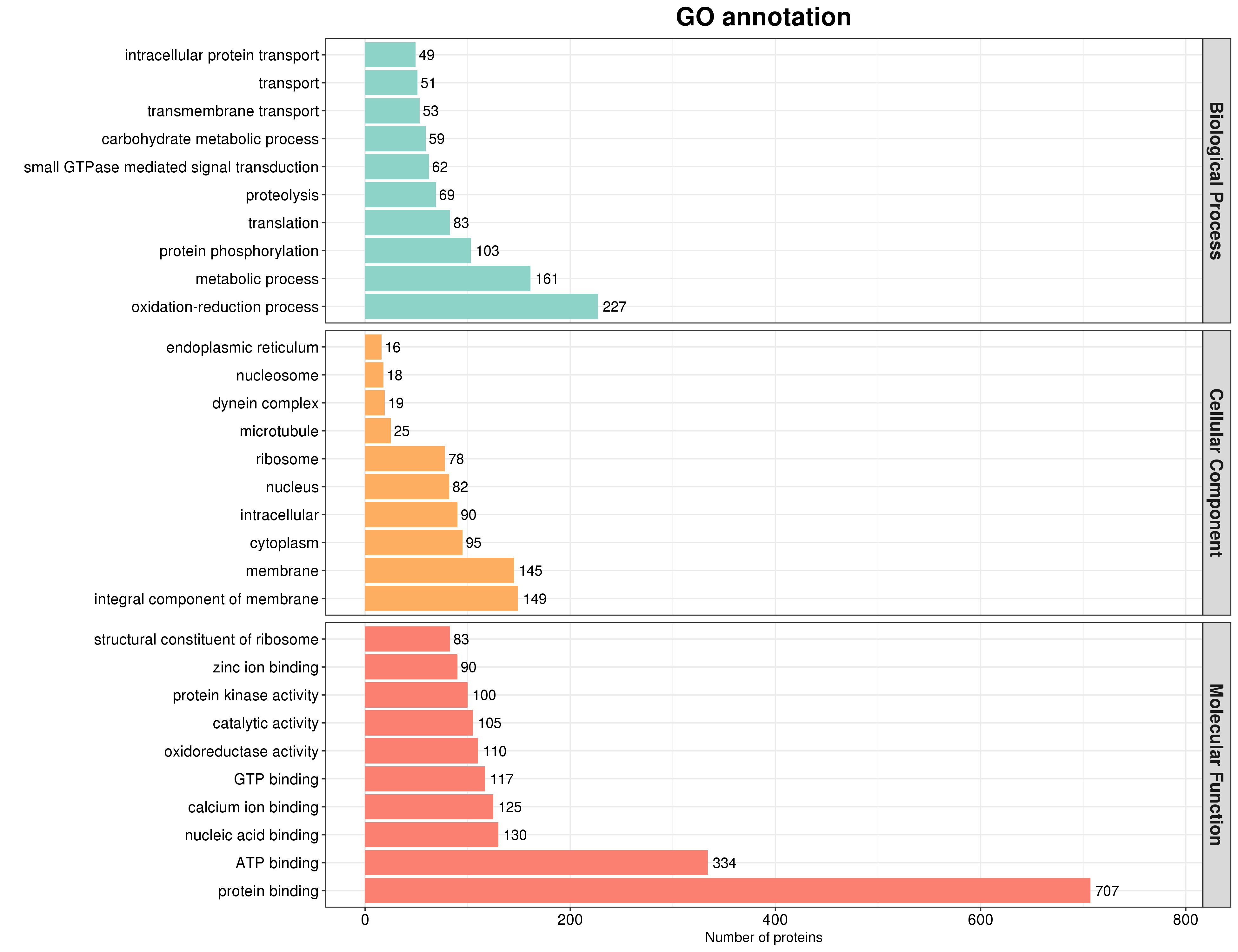

Supplement: S2 Fig — (PNG) [file pone.0301884.s002.png]

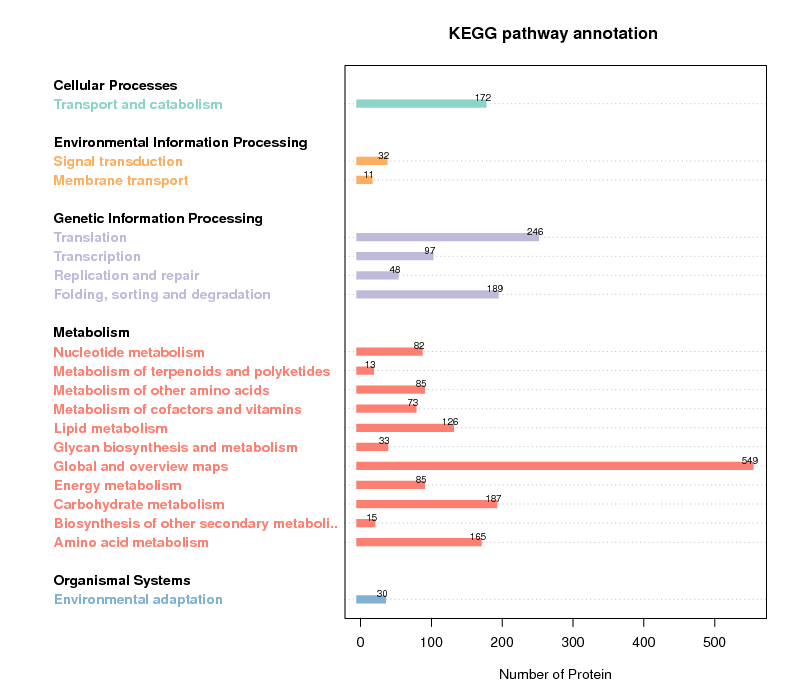

Supplement: S3 Fig — (PNG) [file pone.0301884.s003.png]

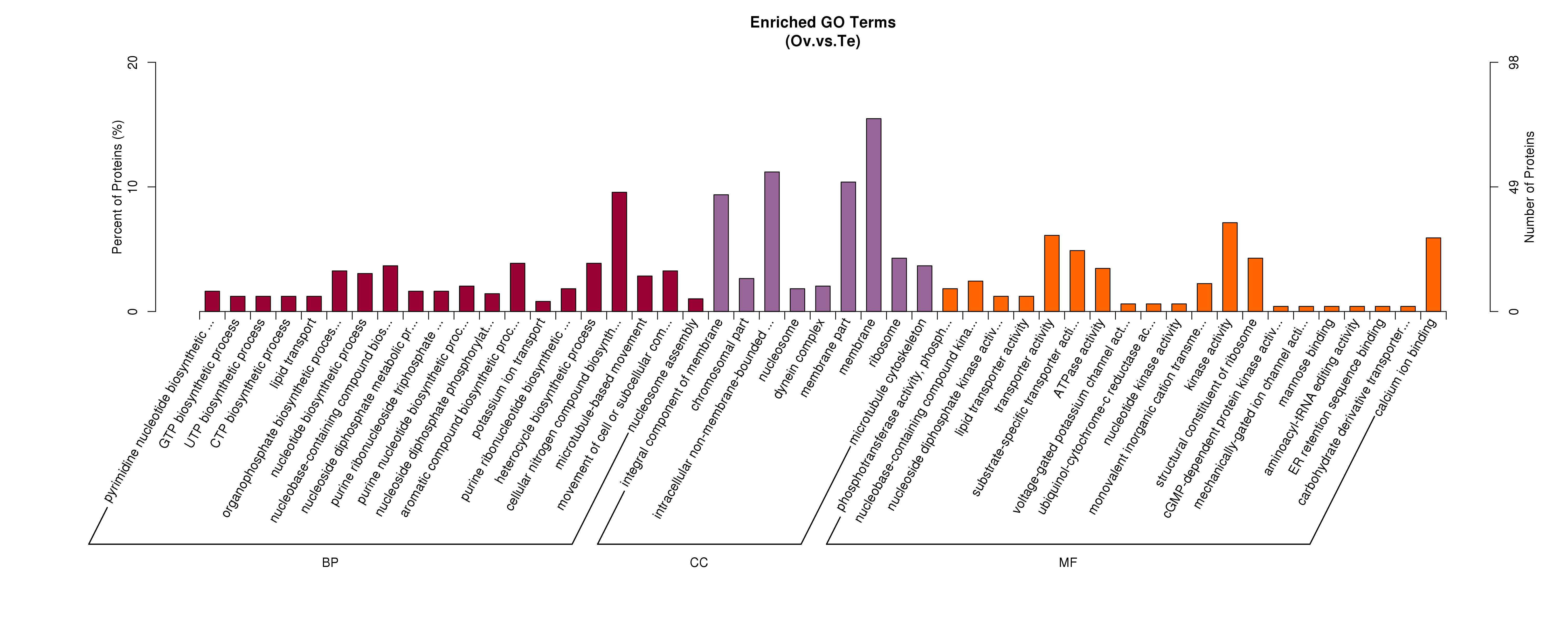

Supplement: S4 Fig — (PNG) [file pone.0301884.s004.png]
